# Report of Two Cases of Diptheria, with Remarks

**Published:** 1859-09

**Authors:** DeLaskie Miller

**Affiliations:** Prof. of Obstetrics in Rush Medical College


ARTICLE II.

### REPORT OF TWO CASES OF DIPTHERIA, WITH REMARKS.

(Read before the Chicago Academy of Medical Sciences.)

By DeLaskie Miller, M. D. Prof. of Obstetrics in Rush Medical College.

A disease of the throat, known as "membranous sore throat," and designated also "diptheria," has prevailed of late to a considerable extent in different sections of this country, and also in various parts of Europe, assuming there, as in some parts of the United States, a most malignant and fatal form, but which, as I believe, has not been met with in this city until quite recently. The following cases, presenting the characteristic appearances and pursuing the usual course of this disease, may be of some interest at the present time.

I was consulted, at my house, July 13th, in regard to the illness of the little daughter of Mrs. W., aged about 8 years, who was "suffering from sore throat." Learning that the bowels were costive and that she had some fever (I did not apprehend an attack of scarlatina, for I had attended the child for that disease two years before), I advised a saline cathartic, to be followed by a full dose of Dover's powder.

Upon visiting the patient next day, which was the first time that I examined the case, I learned that she had been affected with soreness of the throat and painful deglutition for several days, accompanied by the usual symptoms characteristic of an onset of fever, such as frequent chills, alternating with flushes of heat, with darting pains through the back and limbs, pain in the head, with frequent pulse. The submaxillary glands were considerably swelled and tender to the touch; the tongue was covered with a thick, tenacious, yellowish coat; the fauces were of a deep red color and slightly edematous, and were, with the tonsils, partially covered with numerous small patches of a fibrinous exudation, which presented a dirty, yellowish-white color; the gums were also considerably swelled. I prescribed Dover's powder, as occasion required, to control pain and restlessness; also, chlorate of potassa 4 grs. every fourth hour, in solution, and the following as a gargle, to be diluted when used:

Liq. soda chlorinat.; also the following gargle, to be used frequently:

| | | |
|---|---|---|
| ℞ Plumbi. acet., | grs. xx. | |
| Morph. " | " iv. | |
| Glycerine, | ℥ij. | |
| Aq. rosa, | ℥ij. | M. |

*15th, a.m.* Appears much the same as yesterday; the glands rather larger and more tender; the pharynx quite covered with the fibrinous exudation. Much relief has been experienced by the use of the gargle, which I ordered repeated more frequently, and continue medicine as before. I also urged the importance of giving nourishment, which the patient was averse to taking, on account of the pain occasioned by the act of deglutition.

*18th.* A slight amelioration of all the symptoms; continue treatment.

*20th.* Still improving; appetite returning.

From this date she continued to convalesce, though slowly, till to-day (August 1st). She appears to have quite recovered, save some degree of debility.

Mr. C. M. consulted me, July 21st, on account of sore throat, which came on three or four days before, and was getting worse rapidly, causing considerable pain, especially during deglutition. I found the tonsils slightly swelled, and preternatural redness and tumefaction through the pharynx, with small patches of diptheritic membrane on the tonsils and fauces; the tongue thickly coated and yellow. Except the local symptoms in the throat, the patient professes himself as feeling quite well. I applied a strong solution of nitrate of silver to the affected part, and ordered a cathartic, and the application of spirits of turpentine externally to the throat.

*22nd.* The patient has considerable fever, and suffers more from the affection of the throat; the exudation has become thick and continuous, forming a pseudo-membrane, covering the pharynx; the uvula very much swelled and elongated, causing the greatest annoyance, rendering excision of a portion of it necessary; the bleeding, which was considerable, afforded slight but immediate relief. I prescribed chlorate of potassa and morphia in solution internally, and the solution of chlorinated of soda; also, acetate of lead in glycerine, as a gargle, to be used frequently.

After about the third day after I first saw him, the diptheritic membrane began to separate, and was gradually removed in considerable patches, leaving the parts below intensely red and slightly roughened, and from the tongue the papilla projected in long acuminated points. At this stage the patient felt considerable prostration, and his pulse becoming soft and weak, I gave the following, in addition to the medicine before prescribed:

| | | |
|---|---|---|
| ℞ | Quinia sulph., | ℈iss. |
| | Acid sulph. arom., | ʒij. |
| | Tr. gentian co., | ℥j. |
| | Syr. raspberry, | ℥j. |

M. S. Take a teaspoonful every sixth hour.

The progress of his convalescence from this time has been continuous, characterized, however, by unusually slow return of strength, which even now (August 1st) is not complete.

I might add other cases, but as they differ in no essential particular, either in the symptoms, course, treatment or termination, they would unnecessarily prolong this paper.

There are several diseases having some symptoms in common with diptheria, but differing, as I conceive, so essentially in their nature, as to be distinguished without difficulty, viz., pharyngitis, inflammation of the tonsils, croup, and the angina of scarlatina.

That it is not pharyngitis, as usually met with, or as modified by the depressing influences that aggravate disease among the poor in cities, or from impaired constitutions of the patients, is manifest from the course of the symptoms and by the appearance of the pseudo-membrane. The patients which I have attended with this disease have been in good circumstances in life, and reside in healthy localities in the city. The direct depressing influence of diptheria upon the system is also much greater than would be expected from pharyngitis.

The same circumstances also distinguish it from tonsilitis, so far as they relate to the course of the symptoms and the exudation of the fibrinous membrane, which is not a concomitant of inflammation of the tonsils.

The membranous exudation is met with in croup, but in croup the parts first affected are the trachea and the larynx, and the membrane is confined to the trachea, or reaches the pharynx only by extension from below; whereas, in diptheria, the parts especially involved are those concerned in deglutition, and the membrane extends from above downwards, and has uniformly presented the appearance of having a feebler organization than the membrane of croup.

From the angina of scarlatina, diptheria may be distinguished, first, by the presence of the membrane, and, second, by the absence of the eruption, and also, in the cases which have come under my observation, by the fact, that the patients were not subject to scarlet fever.

For these reasons, and others which will present themselves to the notice of every practitioner who may have occasion to treat it, we have sufficient grounds for treating diptheria as a distinct affection.

